# Up-regulation of cell cycle arrest protein BTG2 correlates with increased overall survival in breast cancer, as detected by immunohistochemistry using tissue microarray

**DOI:** 10.1186/1471-2407-10-296

**Published:** 2010-06-16

**Authors:** Elin Möllerström, Anikó Kovács, Kristina Lövgren, Szilard Nemes, Ulla Delle, Anna Danielsson, Toshima Parris, Donal J Brennan, Karin Jirström, Per Karlsson, Khalil Helou

**Affiliations:** 1 Department of Oncology, Institute of Clinical Sciences, Blå stråket 2, University of Gothenburg, SE-413 45 Göteborg, Sweden; 2 Pathology section, Department of Pathology, Sahlgrenska University Hospital, Blå stråket 2, University of Gothenburg, SE-413 45 Göteborg, Sweden; 3 Department of Oncology, Barngatan 2:2, Lund University Hospital, University of Lund, SE-221 85 Lund, Sweden; 4 Division of Clinical Cancer Epidemiology, Department of Oncology, Sahlgrenska Academy, University of Gothenburg, Sweden; 5 UCD School of Biomolecular and Biomedical Science, UCD Conway Institute, University College Dublin, Belfield, Dublin 4, Ireland; 6 Department of Laboratory Medicine, Center for Molecular Pathology, Lund University, University Hospital MAS, Malmö, Sweden; 7 Department of Oncology, Sahlgrenska University Hospital, Blå stråket 2, University of Gothenburg, SE-413 45 Göteborg, Sweden

## Abstract

**Background:**

Previous studies have shown that the *ADIPOR1*, *ADORA1*, *BTG2 *and *CD46 *genes differ significantly between long-term survivors of breast cancer and deceased patients, both in levels of gene expression and DNA copy numbers. The aim of this study was to characterize the expression of the corresponding proteins in breast carcinoma and to determine their correlation with clinical outcome.

**Methods:**

Protein expression was evaluated using immunohistochemistry in an independent breast cancer cohort of 144 samples represented on tissue microarrays. Fisher's exact test was used to analyze the differences in protein expression between dead and alive patients. We used Cox-regression multivariate analysis to assess whether the new markers predict the survival status of the patients better than the currently used markers.

**Results:**

BTG2 expression was demonstrated in a significantly lower proportion of samples from dead patients compared to alive patients, both in overall expression (*P *= 0.026) and cell membrane specific expression (*P *= 0.013), whereas neither ADIPOR1, ADORA1 nor CD46 showed differential expression in the two survival groups. Furthermore, a multivariate analysis showed that a model containing BTG2 expression in combination with HER2 and Ki67 expression along with patient age performed better than a model containing the currently used prognostic markers (tumour size, nodal status, HER2 expression, hormone receptor status, histological grade, and patient age). Interestingly, BTG2 has previously been described as a tumour suppressor gene involved in cell cycle arrest and p53 signalling.

**Conclusions:**

We conclude that high-level BTG2 protein expression correlates with prolonged survival in patients with breast carcinoma.

## Background

Breast cancer is the most common malignancy among women, and accounted for approximately 1.15 million new cases and 411,000 deaths worldwide in 2002 [1]. During the last decade, the survival rate for breast cancer patients has increased dramatically due to earlier detection and new treatment protocols [2]. Presently, various clinical and pathological markers including axillary lymph node status, hormone receptor status, histological grade, tumour size, patient age, HER2 expression and vascular invasion are used to predict breast cancer prognosis and provide accurate treatment [3]. However, these markers are insufficient and approximately 20 to 30% of breast cancer patients will die from the disease within five years of diagnosis [4]. It is, therefore, of great importance to identify novel molecular markers to further refine prognosis and response to treatment. Gene expression analysis has been used to develop gene expression signatures that predict clinical outcome in breast cancer patients [5-9]. Previously, we analysed breast tumours from lymph node-negative patients using gene expression microarray and array-CGH to identify genes with altered levels of expression and aberrant chromosomal regions revealing prognostic values [7,10]. By integrating the expression and array-CGH results, 27 genes were identified which differed significantly (*P *< 0.05) in both gene expression and DNA copy numbers between deceased patients and 10-year survivors [10]. Based on their involvement in breast cancer and the availability of commercial antibodies, the *ADIPOR1*, *ADORA1*, *BTG2 *and *CD46 *genes were selected among the 27 previously identified genes to further investigate the association of protein expression levels to overall patient survival. In the present investigation, protein expression was analysed by immunohistochemistry on tissue microarrays in an independent cohort of breast cancer patients, and correlated to 5-year survival.

## Methods

### Patients and tissue microarray construction

The breast cancer samples were obtained from 144 patients undergoing surgical resection at Malmö University Hospital, Malmö, Sweden, between 2001 and 2002. One patient lacked five years follow-up time resulting in the exclusion of this sample from the 5-year survival analysis, although not from the multivariate analysis. The 5-year survival analysis was performed based on overall survival, including 111 samples from alive and 32 samples from dead patients. Further clinical information is compiled in Table 1. Tissue microarrays (TMAs) containing duplicate 1.00 mm cores from each tumour were constructed as previously described [11]. The utilization of the tumour material for research purposes was approved by regional ethical committees in Lund, Sweden.

**Table 1:** Clinicopathological features of the 144 breast tumour specimens included in this study

	deceased patients	5 year survivors	lack 5 years follow-up	Total
Median age at diagnosis	77	63	75	65
				
Recurrence free for 5 years				
yes	0	102	0	102
no	16	7	1	24
missing	16	2	0	18
Total	32	111	1	144
				
Type				
ductal	26	77	1	104
lobular	4	23	0	27
tubular	1	6	0	7
medullary	1	2	0	3
missing	0	3	0	3
Total	32	111	1	144
				
Size				
median (mm)	27	19	27	20
				
20 mm and below	11	62	0	73
above 20 mm	21	49	1	71
Total	32	111	1	144
				
Nodal status				
positive	17	38	1	56
negative	10	63	0	73
missing	5	10	0	15
Total	32	111	1	144
				
Estrogen receptor status				
positive	23	101	1	125
negative	9	10	0	19
Total	32	111	1	144
				
Progesterone receptor status				
positive	15	85	0	100
negative	17	26	1	44
Total	32	111	1	144
				
Her2 status				
positive	7	6	0	13
negative	22	101	0	123
missing	3	4	1	8
Total	32	111	1	144

### Immunohistochemistry (IHC)

The expression of ADIPOR1, ADORA1, BTG2 and CD46 proteins was investigated using IHC. Prior to hybridisation to the tissue microarrays, antibodies corresponding to the selected genes were optimised on paraffin-embedded sections of breast tumours. After deparaffinisation in Xylene, the tissue microarrays were autoclaved for at least one hour in buffer S1699 or S2367 (Dako Norden A/S, Denmark) or Borgs Decloaker pH9 buffer solution (Biocare Medical, CA, USA) (Table 2). The immunohistochemical staining was performed in an automated immunostainer (TechMate 228 500 Plus; Dako Norden A/S, Denmark). The TMA sections were incubated with the different antibodies at a dilution of 1:300 for ADIPOR1 (Phoenix Pharmaceuticals, Inc, CA, USA), 1:500 for ADORA1 (Genway Biotech, Inc, CA USA), 1:1000 for BTG2 (Genway Biotech, Inc, CA, USA), and 1:40 for CD46 (BD Biosciences, New Jersey, USA); (Table 2). The antibodies were visualised with the EnVision (K5007, Dako Norden A/S, Denmark) or LSAB (K5001, Dako Norden A/S, Denmark) visualization system according to the manufacturer's instructions (Table 2). EnVision uses a secondary antibody against both rabbit and mouse that is directly labelled with HRP (horseradish peroxidase) reacting with DAB, whereas LSAB uses a secondary antibody against rabbit and mouse, labelled with biotin, then streptavidin-HRP is added and the staining is done with DAB. The TMA sections were then washed in water, dehydrated in an alcohol gradient followed by Xylen treatment, and mounted.

**Table 2:** Technical data of the laboratory procedure for each specific antibody

Antibody	Antibody dilution	Manufacturer	Catalogue number	Type of antibody	Time of incubation (RT)	Pre-treatment buffer	visualization system
AdipoR1	1:300	Phoenix Pharmaceuticals	H-001-44	Rabbit polyclonal	30 min	Borgs decloaker	LSAB
Adora1	1:500	Genway Biotech	18-461-10001	Rabbit polyclonal	30 min	Borgs decloaker	LSAB
BTG2	1:1000	Genway Biotech	18-003-42396	Rabbit polyclonal	30 min	S2367	Envision
CD46	1:40	BD Biosciences	555948	Mouse monoclonal	30 min	S1699	Envision

### Evaluation of IHC

The immunostained tissue microarray sections were analysed by a pathologist (AK). CD46 protein is a cell membrane protein, while ADIPOR1 and ADORA1 are cytoplasm proteins. The subcellular location of BTG2 has varied in previous publications, and in this study, the cytoplasm and cell membrane were stained. The cell membrane staining intensities were graded as no expression (0), low expression (+), moderate expression (++), and high expression (+++). The expression of the cytoplasm proteins was also graded from 0-3 (ranging from no expression to high expression). The proportion of tumour cells expressing membrane and/or cytoplasm protein was determined. In the evaluation of ADIPOR1, ADORA1 and CD46, any level of sample staining was considered positive. For the BTG2 protein, moderate to strong staining of at least 50% of the cells was required for the sample to be scored as positive.

### Statistical analysis

The difference in expression of the four proteins between tumours from alive and dead patients was tested using two-tailed Fisher's exact test. Kaplan-Meier survival curves were produced using the SPSS version 16 software to demonstrate the difference in overall survival between overall BTG2-positive and overall BTG2-negative samples, as well as BTG2 membrane-positive and BTG2 membrane-negative samples. Significant differences between the curves were compared using the Breslow-Wilcoxon test [12]. Additionally, a multivariate analysis (Cox-regression) was performed to evaluate the clinical significance of using current prognostic markers (tumour size, nodal status, HER2 expression, hormone receptor status, histological grade, and patient age) versus using a model containing the proposed markers HER2 expression, patient age, and increasingly used marker Ki67 in combination with BTG2 expression. To cope with the reduced validity of the scientific inference due to misrepresentation we used Multiple Imputation by Chained Equations. The effect of the markers on survival probability was modelled by Proportional Hazards Model. Variable selection was based on a combination bootstrap and information theory approach [13]. As an external validating measure we used time-dependent AUC and Concordance index (C-index) [14]. The time-dependent AUC characterises the temporal changes in predictive accuracy. Concordance index offers an easy way to interpret global accuracy measure that varies between 0.5 and 1. A concordance index of 1 means that with 100% precision we can rank the patient's survival time given the recorded marker information. If the concordance index converges to 0.5 the ranking of survival times becomes more and more driven by chance, and becomes completely random at 0.5. To quantify the impact of a single marker on the predictive accuracy we removed one marker at the time from the final model and refitted a Proportional Hazards Model and re-estimated the C-index. An estimation of the correlation between expression of the different proteins were performed using Pearson correlation.

## Results

**Figure 1:**
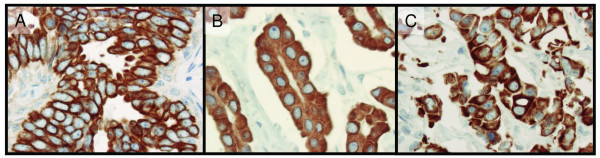
**Sub-cellular location of the BTG2 protein in this study**. Brown colour represents BTG2 staining. BTG2 protein immunohistochemistry staining using tissue microarrays show exclusive cell membrane specific expression (A), exclusive cytoplasm expression (B), and both cell membrane and cytoplasm expression of BTG2 in the same sample (C).

**Figure 2:**
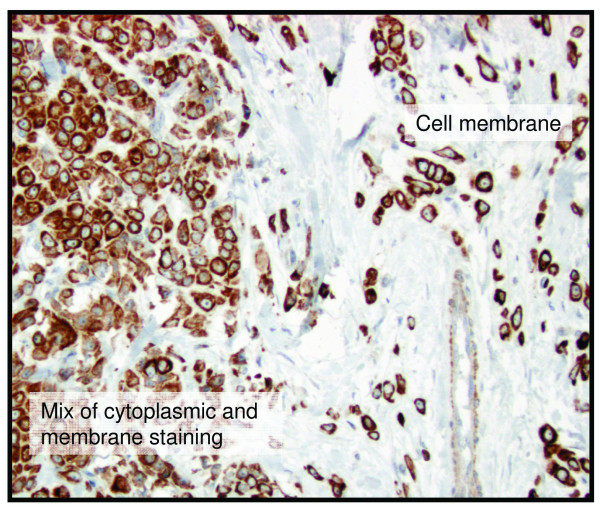
**Various sub-cellular location of the BTG2 protein in even within one sample**. One TMA sample showing cytoplasm and cell membrane expression of BTG2 in a part of the sample and exclusively membranous expression in another part of the sample. Brown colour indicates BTG2 staining.

**Figure 3:**
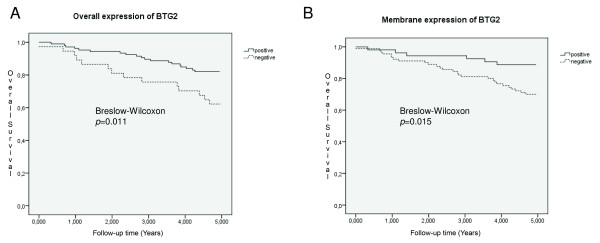
**Kaplan-Meier survival curves illustrating the effect of BTG2 expression**. The Kaplan-Meier curves show the difference in survival between patients with tumours that revealed any BTG2 expression and patients whose tumours did not (A), as well as the difference in survival between patients with tumours that revealed cell membrane specific BTG2 expression and patients whose tumours did not (B). Solid lines represent patients whose tumours expressed BTG2 and dashed lines represent patients whose tumours did not. The p-values for the difference between the curves were calculated using a generalized Wilcoxon test.

**Figure 4:**
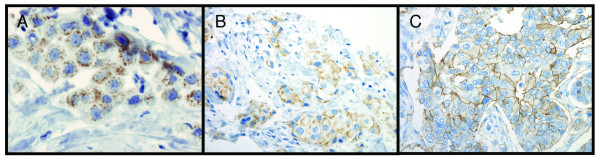
**Sub-cellular location for samples staining positive for ADIPOR1, ADORA1 and CD46**. Brown colour represents staining of the specific protein. Immunohistochemistry staining performed for these proteins on tissue microarrays show staining of ADIPOR1 (A) and ADORA1 (B) as granular staining in the cytoplasm, and expression of CD46 concentrated to the cell membrane (C).

Of the 144 specimens present in the tissue microarray, 136-141 were interpretable for protein expression (Table 3). Excluded samples had few tumour cells, large tissue loss or affluence of necrotic tissue. BTG2 was expressed in both the cytoplasm and the cell membrane (Figure 1). In one sample, BTG2 showed expression in the cytoplasm and the membrane in one area and was expressed in the membrane exclusively in another area (Figure 2). The proportion of BTG2 protein expression was higher in tumours from 5-year overall survivors than among the tumours from deceased patients. The overall expression differed significantly between alive and dead patients (*P *= 0.026), although there was a stronger association with membrane specific expression (*P *= 0.013) (Table 3). Kaplan-Meier curves visualize the difference in overall survival between patients with tumours positive versus negative for overall and cell membrane specific BTG2 expression in Figure 3. Moreover, the difference between the curves was significant using the Breslow-Wilcoxon test [12] for both overall BTG2 (*P *= 0.011) and cell membrane expression (*P *= 0.015). Cytoplasm or cell membrane expression of BTG2 was observed in 78% of the samples and cell membrane specific expression in 39% of the samples. None of the remaining three analysed proteins (ADORA1, ADORA1 and CD46) showed a statistically significant difference in expression between alive and dead patients in this study (Table 3). The ADIPOR1 protein was expressed in the cytoplasm in 18% of the samples. The majority of the positive samples showed primarily granular staining (Figure 4a). Approximately 24% of the samples were positive for ADORA1 staining, also displaying primarily granular staining in the cytoplasm (Figure 4b). Fourteen percent of the samples expressed CD46 in the cell membrane (Figure 4c).

**Table 3:** Difference in protein expression between tumours from 5-year overall survivors and tumours from deceased patients

	Dead patients		Alive patients			
**Protein**	**Protein expression positive (%)**	**Protein expression negative (%)**	**Protein expression positive (%)**	**Protein expression negative (%)**	**not available (No of samples)**	***P*-value dead vs. alive patients**

AdipoR1	26	74	17	83	3	0.29
						
Adora1	30	70	23	77	8	0.47
						
BTG2	61	39	82	18	6	0.026*
-membrane only	19	81	44	56	6	0.013*
-cytoplasm only	52	48	68	32	6	0.14
						
CD46	16	84	14	86	4	0.77

*P*-values were calculated using a two-tailed Fisher's exact test. The samples designated as not available had few tumour cells, large tissue loss or affluence of necrotic tissue.

**Figure 5:**
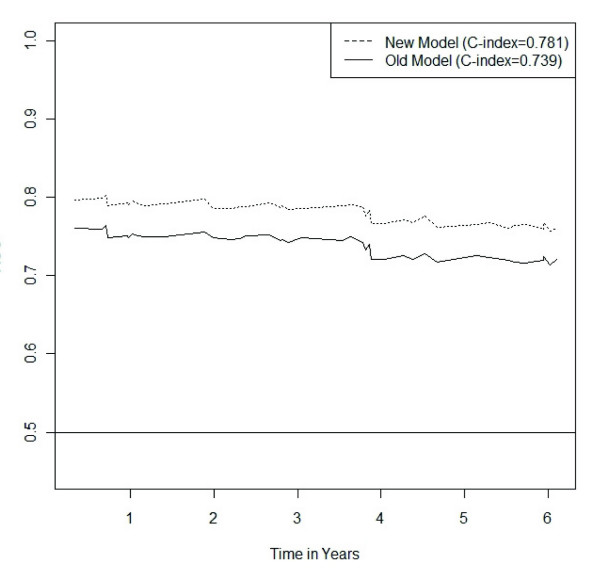
**Variation of the predictive power depending on survival time**. The new Cox-regression model containing BTG2 expression, HER2 expression, patient age and Ki67 expression performed better, i.e. revealed higher prediction accuracy, than a Cox-regression model containing the currently used prognostic markers that gained statistical significance (HER2 expression, and patient age). This difference in predictive power was stable, independent of survival time.

**Figure 6:**
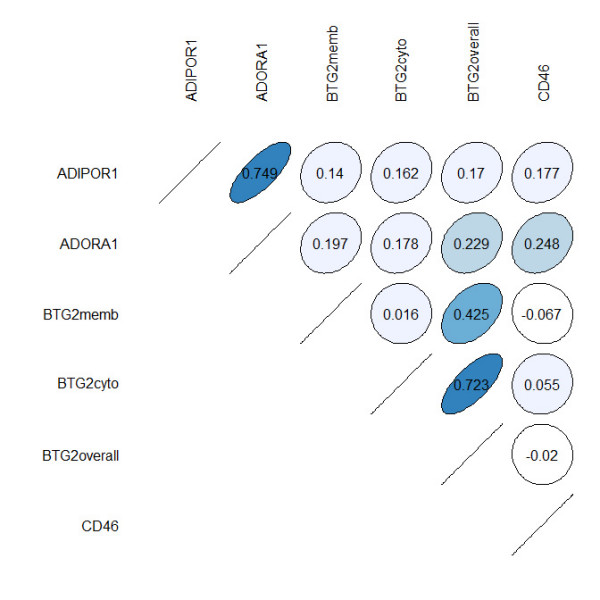
**Correlation plot for the expression of the analyzed proteins**. The strongest correlations were found between ADIPOR1 and ADORA1, as well as BTG2 overall expression and BTG2 cytoplasm expression. The course of the circle indicates whether the correlation is positive or negative.

The multivariate analysis showed that the model containing BTG2 expression had better predictive power than the model built on current classical pathological markers (Table 4). The BTG2 model revealed a C-value of 0.781 compared to the slightly lower C-value of 0.772 for the model of currently used markers. If only the markers displaying statistical significance are used from the model of current prognostic markers (HER2 expression and patient age), a C-value of 0.739 was achieved. Figure 5 shows the classification accuracy of a Cox-regression model based on the current markers compared to the classification accuracy of a new model based on the new markers considered in the present study. Over the whole time-span considered the new markers offer superior classification accuracy. For both the new markers and the old ones the classification accuracy shows a slight decreasing trend with time. The strongest correlation of protein expression were between ADIPOR1 and ADORA1 (*k *= 0.749) and between BTG2 overall expression and BTG2 cytoplasm expression (*k *= 0.723) (Figure 6).

**Table 4:** The effect on the survival status of currently used markers compared to BTG2 expression

A	Odds Ratio	95%CI	P-value	C-index
Age	1.058	1.031; 1.087	< 0.0001*	0.711
BTG2 both	0.338	0.336; 0.339	< 0.0001*	0.768
BTG2 cytoplasm	0.699	0.697; 0.700	< 0.0001*	0.768
BTG2 membrane	0.980	0.977; 0.983	< 0.0001*	0.768
HER2	3.331	3.248; 3.416	< 0.0001*	0.768
Ki67	2.441	1.202; 4.956	0.013*	0.758

				
**B**	**Odds Ratio**	**95%CI**	**P-value**	**C-index**

Age	1.058	1.032; 1.085	< 0.0001*	0.696
HER2	2.668	2.640; 2.696	< 0.0001*	0.761
Histological grade 2	0.954	0.294; 3.094	0.938	0.770
Histological grade 3	1.463	0.432; 4.949	0.539	0.770
Hormone receptor status	0.567	0.248; 1.296	0.179	0.767
Nodal Status	1.673	0.823; 3.398	0.154	0.768
Tumour size	1.569	0.739; 3.330	0.240	0.765

A Cox-regression multivariate analysis was performed to test the effect on survival status of the markers used in current clinical praxis and the changes in the model predictive power induced by the removal of a single marker. The predictive power of the full new model as measured by the C-index is 0.781 (A). The C-index for the old model is 0.772 (B), and the model with only the statistically significant variables in the old model has a predictive power of 0.739.

## Discussion

In the present investigation, the expression of four proteins (ADIPOR1, ADORA1, BTG2 and CD46) was studied by IHC using tissue microarrays. The aim was to evaluate the association between protein expression of these four genes and 5-year overall survival in breast cancer patients. Protein expression of BTG2 was found to be significantly more prevalent in tumours from alive patients compared to tumours from dead patients. Furthermore, a multivariate analysis showed that a model containing BTG2 expression in combination with HER2 expression, patient age and Ki67 expression performed better, i.e. revealed a higher prediction accuracy, than a model containing the currently used prognostic markers (tumour size, nodal status, HER2 expression, hormone receptor status, histological grade, and patient age). In this model, BTG2 expression as well as HER2 expression and patient age were highly significant (*P *< 0.0001), whereas Ki67 expression displayed a lower significance (*P *= 0.013). These results further strengthen that BTG2 expression could be a useful complement to the currently used markers, and in addition, suggest Ki67 expression as a useful marker of breast cancer survival. This is the first report of a large quantitative analysis demonstrating that BTG2 expression is associated with breast cancer patient survival.

A portion of the samples demonstrated cell membrane specific expression only, several showed only cytoplasm expression, and many of the samples showed expression in both the cell membrane and the cytoplasm (Figure 1). One sample showed distinct expression of BTG2 in the cytoplasm and cell membrane in one area and in another area BTG2 was exclusively expressed in the cell membrane (Figure 2). In previous studies of BTG2, the sub-cellular location of the protein was diverse. The protein has been reported to be located in the cytoplasm [15-17] and in the nucleus [18]. Immunostained lung, kidney and small intestine tissue display cell membrane expression of BTG2 in one report [19]. In the present investigation, we observed cytoplasm and cell membrane expression, but no nuclear expression. The various sub-cellular locations of BTG2 could indicate that the protein is expressed in different cellular compartments during altering conditions, such as cell cycle phase, differentially expressed in diverse tissue types, or due to varying specificity of different antibodies.

The *BTG2 *gene is located at the 1q32 chromosomal region, which was gained in a significantly higher proportion of 10-year survivors than in deceased lymph node-negative breast cancer patients in a previous study [10]. The gene belongs to the structurally homogeneous BTG family of which five genes have been identified in human, *BTG1*, *BTG2*, *BTG3*, *Tob *and *Tob2*. The BTG2 protein is highly conserved and shares 94% homology with the murine equivalent [20]. BTG2 is a tumour suppressor gene [21-23] which is directly regulated by p53 and involved in p53-mediated response to DNA damage [24]. According to the literature, BTG2 is involved in cell cycle arrest in the transition from G_1 _to S phase [25,26]. In addition, BTG2 can regulate G_2 _to M cell cycle arrest independent of p53 [27,28]. BTG2 is known to mediate chemotherapy induced apoptosis in cancer cells [29-31] and a study by Lim *et al*. indicates that BTG2 enhances cancer cell death by accumulation of H_2_O_2 _[32].

Down-regulation of BTG2 has been observed in several cancer types such as prostate cancer, breast cancer and gliomas [17,18,33]. In this study 78% of the breast cancer samples showed moderate to high expression of BTG2 in the majority of tumour cells. Nevertheless, BTG2 was significantly down-regulated in tumours from dead patients compared to tumours from alive patients, both in overall expression and cell membrane specific expression. This finding suggests that high total BTG2 or specific cell membrane expression may contribute to a prolonged survival. A previous study analysed BTG2 protein expression and correlated decreased nucleus expression to a more aggressive phenotype of breast cancer, although they did not detect a significant difference in survival [34]. This discrepancy could be due to the use of different BTG2 antibodies since Kawakubo *et al*. detected staining predominantly in the nucleus. However, our finding supports the theory that down-regulation of BTG2 contributes to a more malignant behaviour of the cells. BTG2 is therefore a promising prognostic marker in breast cancer.

The proteins ADIPOR1, ADORA1 and CD46 did not demonstrate differences in expression between tumours from alive and dead patients. High mRNA levels of ADIPOR1 have earlier been associated with lower risk of breast cancer [35]. Previously, Mirza *et al*. speculated that increased expression of ADORA1 may contribute to tumour cell growth and decreased apoptosis in breast tumour cells [36]. The intensity of CD46 expression has been negatively correlated with histological grade and type, tumour size, and tumour recurrence but not to overall survival [37], which is supported by the results from this study. These proteins may still be involved in breast cancer progression, although no significant difference was seen in expression between alive and dead patients in the current investigation. The strongest correlations in protein expression were between ADIPOR1 and ADORA1, as well as BTG2 overall expression and BTG2 cytoplasm expression. The BTG2 correlation was expected since BTG2 overall expression is a combination of BTG2 cytoplasm expression and BTG2 membrane specific expression. The correlation between ADIPOR1 and ADORA1 is however difficult to explain since they to our knowledge do not interact, although both were expressed in similar proportions of the samples in this study (Table 3), and they are located at 1q32, within 300 000 base pairs from each other. This correlation might be interesting for further investigation.

## Conclusions

We conclude that high BTG2 expression levels correlate with prolonged breast cancer survival. Furthermore, BTG2 protein expression may be used as a prognosticator for breast cancer as well as a possible molecular target in breast cancer treatment. Further studies in independent tumour sets are needed to validate and establish BTG2 protein expression as a prognostic marker.

## List of abbreviations

AUC: Area Under the Receiver Operating Characteristic Curve; C-index: Concordance index; CGH: Compatative Genome Hybridisation; DNA: Deoxyribonucleic Acid; IHC: Immunohistochemistry; TMA: Tissue Microarray.

## Competing interests

The authors declare that they have no competing interests.

## Authors' contributions

KL, EK and AD performed the immunohistochemistry. AK evaluated the immunostained tissue microarrays. EK performed the 5-year survival analysis. SN performed the multivariate analysis. DB performed the molecular analyses (such as ER, PR, HER2) and KJ provided the tissue microarrays as well as the clinical information. EK, UD, AD, TP, PK, KJ and KH interpreted the results and wrote the paper. All authors read and approved the final manuscript. KH was responsible for supervision as well as providing the funding.

## Pre-publication history

The pre-publication history for this paper can be accessed here:

http://www.biomedcentral.com/1471-2407/10/296/prepub

## References

[B1] Parkin DM Bray F Ferlay J Pisani P Global cancer statistics, 2002 CA Cancer J Clin 2005 55 74 108 10.3322/canjclin.55.2.74 15761078

[B2] Berry DA Cronin KA Plevritis SK Fryback DG Clarke L Zelen M Mandelblatt JS Yakovlev AY Habbema JD Feuer EJ Effect of screening and adjuvant therapy on mortality from breast cancer N Engl J Med 2005 353 1784 1792 10.1056/NEJMoa050518 16251534

[B3] Goldhirsch A Wood WC Gelber RD Coates AS Thurlimann B Senn HJ Progress and promise: highlights of the international expert consensus on the primary therapy of early breast cancer 2007 Ann Oncol 2007 18 1133 1144 10.1093/annonc/mdm271 17675394

[B4] Coleman MP Quaresma M Berrino F Lutz JM De Angelis R Capocaccia R Baili P Rachet B Gatta G Hakulinen T Cancer survival in five continents: a worldwide population-based study (CONCORD) Lancet Oncol 2008 9 730 756 10.1016/S1470-2045(08)70179-7 18639491

[B5] van't Veer LJ Dai H van de Vijver MJ He YD Hart AA Mao M Peterse HL van der Kooy K Marton MJ Witteveen AT Gene expression profiling predicts clinical outcome of breast cancer Nature 2002 415 530 536 10.1038/415530a 11823860

[B6] Wang Y Klijn JG Zhang Y Sieuwerts AM Look MP Yang F Talantov D Timmermans M Meijer-van Gelder ME Yu J Gene-expression profiles to predict distant metastasis of lymph-node-negative primary breast cancer Lancet 2005 365 671 679 15721472 10.1016/S0140-6736(05)17947-1

[B7] Karlsson E Delle U Danielsson A Olsson B Abel F Karlsson P Helou K Gene expression variation to predict 10-year survival in lymph-node-negative breast cancer BMC Cancer 2008 8 254 10.1186/1471-2407-8-254 18778486 PMC2559847

[B8] Naderi A Teschendorff AE Barbosa-Morais NL Pinder SE Green AR Powe DG Robertson JF Aparicio S Ellis IO Brenton JD Caldas C A gene-expression signature to predict survival in breast cancer across independent data sets Oncogene 2006 10.1038/sj.onc.1209920 16936776

[B9] Paik S Shak S Tang G Kim C Baker J Cronin M Baehner FL Walker MG Watson D Park T A multigene assay to predict recurrence of tamoxifen-treated, node-negative breast cancer N Engl J Med 2004 351 2817 2826 10.1056/NEJMoa041588 15591335

[B10] Mollerstrom E Delle U Danielsson A Parris T Olsson B Karlsson P Helou K High-resolution genomic profiling to predict 10-year overall survival in node-negative breast cancer Cancer Genet Cytogenet 2010 198 79 89 10.1016/j.cancergencyto.2009.12.012 20362222

[B11] Ryden L Landberg G Stal O Nordenskjold B Ferno M Bendahl PO HER2 status in hormone receptor positive premenopausal primary breast cancer adds prognostic, but not tamoxifen treatment predictive, information Breast Cancer Res Treat 2008 109 351 357 10.1007/s10549-007-9660-2 17636399

[B12] Gehan EA A Generalized Wilcoxon Test for Comparing Arbitrarily Singly-Censored Samples Biometrika 1965 52 203 223 14341275

[B13] Heymans MW van Buuren S Knol DL van Mechelen W de Vet HC Variable selection under multiple imputation using the bootstrap in a prognostic study BMC Med Res Methodol 2007 7 33 10.1186/1471-2288-7-33 17629912 PMC1945032

[B14] Heagerty PJ Zheng Y Survival model predictive accuracy and ROC curves Biometrics 2005 61 92 105 10.1111/j.0006-341X.2005.030814.x 15737082

[B15] Montagnoli A Guardavaccaro D Starace G Tirone F Overexpression of the nerve growth factor-inducible PC3 immediate early gene is associated with growth inhibition Cell Growth Differ 1996 7 1327 1336 8891336

[B16] Varnum BC Reddy ST Koski RA Herschman HR Synthesis, degradation, and subcellular localization of proteins encoded by the primary response genes TIS7/PC4 and TIS21/PC3 J Cell Physiol 1994 158 205 213 10.1002/jcp.1041580125 8263025

[B17] Ficazzola MA Fraiman M Gitlin J Woo K Melamed J Rubin MA Walden PD Antiproliferative B cell translocation gene 2 protein is down-regulated post-transcriptionally as an early event in prostate carcinogenesis Carcinogenesis 2001 22 1271 1279 10.1093/carcin/22.8.1271 11470758

[B18] Kawakubo H Carey JL Brachtel E Gupta V Green JE Walden PD Maheswaran S Expression of the NF-kappaB-responsive gene BTG2 is aberrantly regulated in breast cancer Oncogene 2004 23 8310 8319 10.1038/sj.onc.1208008 15378000

[B19] Melamed J Kernizan S Walden PD Expression of B-cell translocation gene 2 protein in normal human tissues Tissue Cell 2002 34 28 32 10.1054/tice.2001.0220 11989967

[B20] Duriez C Moyret-Lalle C Falette N El-Ghissassi F Puisieux A BTG2, its family and its tutor Bull Cancer 2004 91 E242 253 15381462

[B21] Boiko AD Porteous S Razorenova OV Krivokrysenko VI Williams BR Gudkov AV A systematic search for downstream mediators of tumor suppressor function of p53 reveals a major role of BTG2 in suppression of Ras-induced transformation Genes Dev 2006 20 236 252 10.1101/gad.1372606 16418486 PMC1356114

[B22] Lim IK Lee MS Lee SH Kim NK Jou I Seo JS Park SC Differential expression of TIS21 and TIS1 genes in the various organs of Balb/c mice, thymic carcinoma tissues and human cancer cell lines J Cancer Res Clin Oncol 1995 121 279 284 10.1007/BF01209594 7768965 PMC12201275

[B23] Elmore LW Di X Dumur C Holt SE Gewirtz DA Evasion of a single-step, chemotherapy-induced senescence in breast cancer cells: implications for treatment response Clin Cancer Res 2005 11 2637 2643 10.1158/1078-0432.CCR-04-1462 15814644

[B24] Cortes U Moyret-Lalle C Falette N Duriez C Ghissassi FE Barnas C Morel AP Hainaut P Magaud JP Puisieux A BTG gene expression in the p53-dependent and -independent cellular response to DNA damage Mol Carcinog 2000 27 57 64 10.1002/(SICI)1098-2744(200002)27:2<57::AID-MC1>3.0.CO;2-I 10657898

[B25] Guardavaccaro D Corrente G Covone F Micheli L D'Agnano I Starace G Caruso M Tirone F Arrest of G(1)-S progression by the p53-inducible gene PC3 is Rb dependent and relies on the inhibition of cyclin D1 transcription Mol Cell Biol 2000 20 1797 1815 10.1128/MCB.20.5.1797-1815.2000 10669755 PMC85361

[B26] Lim IK Lee MS Ryu MS Park TJ Fujiki H Eguchi H Paik WK Induction of growth inhibition of 293 cells by downregulation of the cyclin E and cyclin-dependent kinase 4 proteins due to overexpression of TIS21 Mol Carcinog 1998 23 25 35 10.1002/(SICI)1098-2744(199809)23:1<25::AID-MC4>3.0.CO;2-G 9766435

[B27] Ryu MS Lee MS Hong JW Hahn TR Moon E Lim IK TIS21/BTG2/PC3 is expressed through PKC-delta pathway and inhibits binding of cyclin B1-Cdc2 and its activity, independent of p53 expression Exp Cell Res 2004 299 159 170 10.1016/j.yexcr.2004.05.014 15302583

[B28] Hong JW Ryu MS Lim IK Phosphorylation of serine 147 of tis21/BTG2/pc3 by p-Erk1/2 induces Pin-1 binding in cytoplasm and cell death J Biol Chem 2005 280 21256 21263 10.1074/jbc.M500318200 15788397

[B29] Tirone F The gene PC3(TIS21/BTG2), prototype member of the PC3/BTG/TOB family: regulator in control of cell growth, differentiation, and DNA repair? J Cell Physiol 2001 187 155 165 10.1002/jcp.1062 11267995

[B30] Chen JG Yang CP Cammer M Horwitz SB Gene expression and mitotic exit induced by microtubule-stabilizing drugs Cancer Res 2003 63 7891 7899 14633718

[B31] Islaih M Halstead BW Kadura IA Li B Reid-Hubbard JL Flick L Altizer JL Thom Deahl J Monteith DK Newton RK Watson DE Relationships between genomic, cell cycle, and mutagenic responses of TK6 cells exposed to DNA damaging chemicals Mutat Res 2005 578 100 116 16109433 10.1016/j.mrfmmm.2005.04.012

[B32] Lim YB Park TJ Lim IK B cell translocation gene 2 enhances susceptibility of HeLa cells to doxorubicin-induced oxidative damage J Biol Chem 2008 283 33110 33118 10.1074/jbc.M804255200 18840609 PMC2662249

[B33] Calzolari F Appolloni I Tutucci E Caviglia S Terrile M Corte G Malatesta P Tumor progression and oncogene addiction in a PDGF-B-induced model of gliomagenesis Neoplasia 2008 10 1373 1382 following 1382 19048116 10.1593/neo.08814 PMC2586688

[B34] Kawakubo H Brachtel E Hayashida T Yeo G Kish J Muzikansky A Walden PD Maheswaran S Loss of B-cell translocation gene-2 in estrogen receptor-positive breast carcinoma is associated with tumor grade and overexpression of cyclin d1 protein Cancer Res 2006 66 7075 7082 10.1158/0008-5472.CAN-06-0379 16849553

[B35] Kaklamani VG Sadim M Hsi A Offit K Oddoux C Ostrer H Ahsan H Pasche B Mantzoros C Variants of the adiponectin and adiponectin receptor 1 genes and breast cancer risk Cancer Res 2008 68 3178 3184 10.1158/0008-5472.CAN-08-0533 18451143 PMC2685173

[B36] Mirza A Basso A Black S Malkowski M Kwee L Pachter JA Lachowicz JE Wang Y Liu S RNA interference targeting of A1 receptor-overexpressing breast carcinoma cells leads to diminished rates of cell proliferation and induction of apoptosis Cancer Biol Ther 2005 4 1355 1360 16294023 10.4161/cbt.4.12.2196

[B37] Madjd Z Durrant LG Pinder SE Ellis IO Ronan J Lewis S Rushmere NK Spendlove I Do poor-prognosis breast tumours express membrane cofactor proteins (CD46)? Cancer Immunol Immunother 2005 54 149 156 10.1007/s00262-004-0590-0 15378282 PMC11034299

